# Progress in the classification, optimization, activity, and application of antimicrobial peptides

**DOI:** 10.3389/fmicb.2025.1582863

**Published:** 2025-04-23

**Authors:** Zuheng Su, Huajun Yu, Tingting Lv, Qizhou Chen, Hui Luo, Haitao Zhang

**Affiliations:** ^1^School of Ocean and Tropical Medicine, Guangdong Medical University, Zhanjiang, China; ^2^Guangdong Medical University, Zhanjiang, China; ^3^Department of Biochemistry and Molecular Biology, Guangdong Medical University, Zhanjiang, China; ^4^Department of Neurology, Huazhou People's Hospital, Huazhou, China

**Keywords:** antimicrobial peptides, classification, performance optimization, biological activity, application

## Abstract

Antimicrobial peptides (AMPs) come from various sources and exhibit unique antimicrobial properties. Their rapid action, effectiveness, and resistance to resistance development make them promising alternatives to combat antibiotic resistance. In addition to its excellent antibacterial properties, AMPs have superior immunomodulatory, antitumor, and antiviral activities. In recent years, the demand for AMPs has continued to increase in many fields, especially in the medical field, and the prospects are extensive. However, AMPs have the disadvantages of expensive development cost, higher hemolysis, short half-life, susceptibility to degradation by protein hydrolases, low bioavailability, toxic side effects, and other disadvantages, which seriously limit the wide application of AMPs. Therefore, fewer AMPs have been approved for marketing or are undergoing clinical trials. The review covers the period from 2001 to 2025 and provides a detailed discussion by searching databases such as Google Scholar and Web of Science. This paper reviews the progress of research on AMPs sources, structures, optimization strategies, biological activities, mechanisms of action, and applications. In general, the development approaches and the number of new AMPs have increased significantly. The improvement technologies for AMPs high hemolysis, poor stability, low bioavailability and high cost have increased significantly. The development cost of AMPs is still high, but many AMPs have been widely used in clinical, food, livestock, poultry, cosmetics and other fields. This article focuses on the commonly used optimization strategies and main activities of AMPs, aiming to effectively respond to challenges and provide a theoretical basis for expanding their application range.

## 1 Introduction

The widespread use and misuse of antibiotics have led to the rapid development and spread of antibiotic resistance, putting humanity at risk of a future without effective antibiotics. Therefore, many scientists have devoted themselves to finding antibiotic substitutes in the past decades. In exploring new antimicrobial drugs, antimicrobial peptides (AMPs) stand out, showing great application potential, and are considered excellent candidates for antimicrobial drugs. AMPs, also known as host defense peptides, are one of the essential endogenous defense molecules in the body’s innate immune system, which can effectively and rapidly defend against the attack of foreign microorganisms. AMPs are small peptides with broad-spectrum antimicrobial activity, typically with a molecular weight under 10 kDa, and are widely distributed (Chen and Jiang, 2023). Antibiotics often achieve antimicrobial efficacy by binding to microbial molecular receptors or targets. In contrast, AMPs usually exert their antimicrobial activity by rapidly disrupting microbial cell membranes and causing leakage of their contents (Yang et al., 2024a; Zhang et al., 2024b). This rapid and unique mechanism of action makes it difficult for pathogenic microorganisms to develop resistance to AMPs, which is unmatched by existing antimicrobial drugs. Therefore, AMPs can treat various microbial infections, even drug-resistant microbes. For example, AMPs can effectively fight against super bacteria such as *methicillin-resistant Staphylococcus aureus* and *Pseudomonas aeruginosa* (Onaizi and Leong, 2011). In addition to its prominent antimicrobial activity, it also possesses abundant and excellent biological activities, such as antitumor, anti-inflammatory, antioxidant, antiviral, antidiabetic, antiparasitic, spermicidal, pro-angiogenic, and wound-healing, among many other functions (Luo and Song, 2021; Zannella et al., 2022; Miceli et al., 2024). These activities are exerted through a variety of mechanisms. AMPs have been widely used in medicine, food preservation, agricultural disease resistance, environmental sanitation, aquaculture, and other fields (Huan et al., 2020). However, AMPs still face many challenges in their development and application, such as high development cost, short half-life, low stability, low bioavailability, higher hemolysis, toxic side effects, and other disadvantages (Talapko et al., 2022). Only a relatively small number of species of AMPs have been successfully applied in the fields of pharmaceuticals and others. Therefore, more effective strategies are needed to address these challenges and allow more AMPs to be industrialized and marketed.

This review summarizes the sources, structural features, optimization strategies, bioactivities, mechanisms of action, and applications of AMPs. We aim to develop more novel and effective AMPs efficiently, address the challenges, and provide a theoretical basis for expanding their applications.

## 2 Sources of AMPs

AMPs can be obtained from various sources, including plants, animals, microorganisms, and artificial synthesis technology (Satchanska et al., 2024). Today, the AMPs database reports 3,306 natural AMPs, 231 predicted AMPs, and 1,299 synthetic AMPs (https://aps.unmc.edu/).

### 2.1 Natural AMPs sources

Natural AMPs typically range from 10 to 100 amino acids in length. The isolation and purification technology of natural AMPs is not yet mature, with low extraction efficiency, low yield, and high cost. However, it is still the primary source of AMPs (Lyu et al., 2023). This type of AMPs comes from various sources and can be extracted and isolated from animals, plants, and microorganisms. They are usually produced by biological cells through a ribosome synthesis mechanism. A few microbial AMPs can be produced by non-ribosomal synthases, such as polymyxin, bacillus peptide, short peptide, and others (Koehbach and Craik, 2019).

#### 2.1.1 Derived from animals

In the last few decades, almost all animals have been found to produce AMPs (Patyra and Kwiatek, 2023). Animal AMPs come from various sources, including insects, mammals, amphibians, fish, mollusks, crustaceans, etc. Finding novel antibiotics has become much more difficult than before. Many new AMPs from animal sources have been reported in recent years, and we can look for novel AMPs from animals as alternatives to antibiotics. The new animal AMPs are shown in Table 1. AMPs play a key role in the innate immune system of both vertebrates and invertebrates. They are mainly found in phagocytes of tissues such as skin and mucosal epithelium, which can effectively protect against pathogens (Guani-Guerra et al., 2010). Insects and frog skin are the main sources of animal AMPs, and it is also a critical storage library for natural AMPs (Zhou et al., 2024). Animal-derived AMPs were first discovered in cecropins from invertebrates, and later successively found to be secreted by vertebrates (Datta and Roy, 2020). The first animal AMP (phagocytin) was isolated from rabbit leukemia cells in 1956 (Amaning Danquah et al., 2022). In 1981, cecropins were the first animal to have antibiotics separated from silkworm moths, with antitumor and antibacterial activity (Andersson et al., 2003). Cecropins are one of the most intensively studied and characterized families and have become the lead compounds for many synthetic peptides (Andrä et al., 2001). Temporins are one of the shortest AMPs families isolated from the European red frog *Rana temporaria* (Casciaro et al., 2020). This class of peptides is readily accessible by chemical synthesis and most effectively kills Gram-positive bacteria (D'Andrea and Romanelli, 2023). Recently, Wang et al. (2024b) isolated and purified a novel Kunitzin AMP from the skin secretion of the large odorous frog *Odorrana lividia*, which has stronger antibacterial activity than many reported frog Kunitzin peptides. The prominent families of animal-derived AMPs are cecropins, defensins, cathelicidins and histatins (Wang, 2022). It is noteworthy that AMPs tend to be hemolytic, but cecropin P1, which is derived from porcine gastric nematodes, does not cause hemolysis and has a rapid and potent antimicrobial activity (Han et al., 2011).

**Table 1:** Novel AMPs from animal sources.

Name	Source	Physical and chemical properties	Effect	References
CpAMP	*Tachypleus tridentatus*	Structures with alpha helices and irregular convolutions	Exhibited a broad spectrum of antimicrobial activity	Wang et al. (2025)
Kunitzin-OL	*Odorrana livida*	Contains an additional base sequence-KVKF-	Exhibited dual antibacterial and antitrypsin activity	Wang et al. (2024b)
Tc-33	The Chinese tree shrew	Contains 5 acidic glutamate residues	Weak antimicrobial activity	Li et al. (2024a)
Sparamosin	The Mud Crab *Scylla paramamosain*	Contains α-helical structure with hydrophobic residue ratio of 32 ~ 38	Showed potent antifungal activity against *Cryptococcus neoformans*	Chen et al. (2021b)
Knottin peptide named MaK	*Monochamus alternatus*	Contains 56 amino acid residues	Vigorous antibacterial activity and the ability to inhibit pine wood nematodes	Han et al. (2023)
Lc1687	*Larimichthys crocea*	A novel anionic amphiphilic α-helical peptide	Bactericidal and parasiticidal activity	Chen et al. (2023)
Lausporin-1 and Lausporin-2	*Liocheles* Australasia	Contains α-helical amphipathic molecules	Bactericidal activity against methicillin-resistant *staphylococci*	Zhao et al. (2021)
Dermaseptin-SS1	*Phyllomedusa tarsius*	Amphipathic α-helix configuration	Anti-lung cancer effects	Ma et al. (2023)

Despite the attention that animal AMPs have received from researchers, their extraction and purification processes are still challenging. In particular, some natural AMPs need to be extracted from animal tissues or other natural resources, which is costly and may have resource limitations. Therefore, there is a need to focus on chemical synthesis and genetic engineering to reduce the cost of preparation so that they can become the primary means of obtaining AMPs. The oceans have been called the “pharmacy of the new millennium,” but AMPs research on marine-derived AMPs accounts for only 3% of the total AMPs (Wang et al., 2023b). Marine environments are complex and variable, and the molecular backbones of marine animal AMPs are often unique and valuable. Therefore, the rich AMPs resources of marine animals need to be exploited urgently.

#### 2.1.2 Derived from plants

Currently, more than 1,000 types of AMPs are extracted from plants. The main organs of plants can produce AMPs when infected by pathogens, with seeds and roots being the main sources (Lima et al., 2022). Plant AMPs not only inhibit or kill pathogenic microorganisms in plants, but also promote the growth and development of the plant (Li et al., 2021b). This type of AMPs varies greatly in their sources, structures, physicochemical properties, pharmacological effects, and mechanisms of action, making their classification difficult. Most plant AMPs consist of cysteine residues, and we can briefly categorize plant AMPs based on the order of amino acids, cysteine motifs, and disulfide bonding arrangements (Satchanska et al., 2024). Common plant AMPs species include thionins, defensins, lipid transfer proteins, hevein-like peptides, knottin-like peptides, Ib-AMPs, snakins, cyclotides, *α*-hairpinin, and cysteine-rich AMPs (Bin Hafeez et al., 2021; Lima et al., 2022). Table 2 briefly describes the different families of plant AMPs. As can be seen from the table, plant AMPs tend to contain more cysteines and intramolecular disulfide bonds are formed between the cysteines. To some extent, disulfide bonds are necessary for these peptides to exert antimicrobial activity. Phyto-defensins are highly stable cysteine-rich peptides, consisting of 4 to 45 amino acids. Most phytodefensins have broad-spectrum biological activity and are particularly effective against fungi. In recent years, several cysteine-free plant AMPs have also been discovered (Tavares et al., 2012). For example, Rogozhin et al. (2020) extracted nine components from *Equisetum arvense*, seven of which were linear peptides without cysteine and mainly rich in aspartic and glutamic acid. Fewer novel plant AMPs have been reported in recent years. Wang et al. (2023c) isolated and characterized a novel hydrophobic anionic AMP (MOp3) from *Moringa oleifera* seeds, and MOp3 possessed potent antimicrobial activity and negligible hemolytic activity.

**Table 2:** Sources, structural characteristics and activities of different plant AMPs families.

Species	Source	Number of amino acids	Disulfide bonds	Cysteine residues	Biological activity	References
Thionins	Black cumin seeds	46	4	8	Vigorous antifungal and cytotoxic activity	Barashkova et al. (2021)
Defensins	Scots pine seeds	51	4	8	Antibacterial and antifungal effects	Shalovylo et al. (2021)
Lipid transfer proteins	Fennel seeds	91	4	8	Antitumor effect	Megeressa et al. (2020)
Hevein-like peptides	*Triticumkiharae* seeds	44	5	10	One of the most effective antifungal peptides	Odintsova et al. (2009)
Knottin-like peptides	Wheat	53	-	2	Negatively regulates wheat to stripe rust resistance	Guo et al. (2023)
Ib-AMP4	*Impatiens balsamina* seeds	20	2	4	Antibacterial effect	Sadelaji et al. (2022)
Snakins	Potato	63	-	12	Antifungal effect	Das et al. (2021)
Cyclotides	Tissue of *Viola odorata*	33	-	6	Accelerated labor	Narayani et al. (2017) and Aslam et al. (2022)
α-hairpinin	Buckwheat	41	2	4	Antibacterial and antitumor effects	Dunaevsky et al. (2020)
Non-cysteine rich peptides	Potato	7–14	-	0	Antibacterial effect	Rogozhin et al. (2020)

“-” indicates that this information is not mentioned in the literature.

The purification of plant AMPs is similarly complex and time-intensive. To expand the research and development of plant AMPs. We can perform high-throughput screening of AMPs genes in plants to identify AMPs species that are efficiently expressed and easy to purify, thus improving the purification efficiency. The antimicrobial spectrum of plant AMPs is relatively narrow compared to that of AMPs from animal and microbial sources, and their antimicrobial activity is mainly focused on the defence against phytopathogenic bacteria (Benko-Iseppon et al., 2010). In the future, research on the combined use of plant AMPs with other antimicrobials can be increased to create synergistic effects, thus expanding their antimicrobial spectrum.

#### 2.1.3 Derived from microorganisms

Compared with animal and plant AMPs, microbial AMPs can secrete more immunomodulatory molecules (Lin et al., 2023). Microbial AMPs offer several advantages, including diversity, multifunctionality, gene richness, and easy access to raw materials. They also benefit from complex living environments, short production cycles, and large-scale fermentation, making them highly promising for application and market potential.

This type of AMPs was first identified and used in clinical. The short mycobacterial peptide was the first AMP to be used clinically (Yazici et al., 2018). Antimicrobial drugs such as mucins, vancomycin, daptomycin, *ε*-polylysine, and other antimicrobial drugs have also been approved by the U.S. Food and Drug Administration (FDA) (Lin et al., 2023). Bacteria, fungi, actinomycetes, protozoa, parasites, and viruses can produce AMPs, with bacteria and fungi being the primary sources of novel antimicrobial agents (Amaning Danquah et al., 2022). Activity studies of AMPs from various microbial sources are shown in Table 3.

**Table 3:** Activity studies of AMPs from various microbial sources.

Source	Name	Biological activity	References
*Ligilactobacillus salivarius* P1CEA3	Nisin S	High antimicrobial activity	Sevillano et al. (2023)
*Trichoderma atroviride* O1	Peptaibols	Anti-cancer and anti-bacterial effects	Viglas et al. (2021)
The *Glutamicibacter mysorens*	Kinetin-9-ribose and Embinin	Anti-cancer and anti-bacterial effects	Karthik et al. (2023)
Human hepatitis B virus	HBc ARD	Antibacterial effect	Chen et al. (2013)
Marine parasites	Anisaxins	Antibacterial effect	Roncevic et al. (2022)
*Nostoc calcicola*	Cyclic peptide nostophycin	Antibacterial effect	Gupta and Vyas (2021)
Nasal *Staphylococcus lugdunensis* strains	Lugdunin	Antibacterial effect	Zipperer et al. (2016)
*Xanthomonas albilineans*	Albicidin	Antibacterial effect	Lin et al. (2023)
*Rhodococcus opacus* R7	Biosurfactants	Antibacterial effect	Zampolli et al. (2022)
*Bacillus paralicheniformis* NNS4-3	NNS4-3	Antibacterial effect	Sermkaew et al. (2024)
*Lactobacillus casei* HZ1	LHH 1	Anti-cancer and anti-bacterial effects	He et al. (2020)

Bacteriocins are the most common class of microbial AMPs and are peptides synthesized by bacterial or archaeal ribosomes. Bacteriocins specifically kill competing bacteria at low concentrations without harming the host itself (Paiva and Breukink, 2012). In 1928, the first bacteriocin was discovered in *Lactobacillus lactis*, which effectively inhibited the growth of *Lactobacillus bulgaricus* (Rodriguez et al., 2021). It has been found that a larger number of fungi can produce AMPs, such as peptaibols, plectasin, trtesin, eurocin, trypilepyrazinol, and others (Tejesvi et al., 2013). Most extreme microorganisms and endophytes have not yet been explored, which may be an important avenue for mining novel AMPs.

### 2.2 Synthetic AMPs

The preparation of AMPs by biological extraction methods cannot meet the drug needs in various fields. Therefore, many researchers have worked on developing AMPs classes that also consist of positively charged and hydrophobic groups. AMPs can be designed from scratch or synthesized based on existing peptides templates (Guo et al., 2024b). To date, thousands of AMPs have been synthesized synthetically using natural AMPs as templates (Robles-Fort et al., 2021). Amino acids can be sequentially added to template AMPs sequences, allowing for precise modification, analysis, and optimization of these molecules (Lima et al., 2021).

Many AMPs currently undergoing clinical trials are chemically synthesized peptide mimics (Browne et al., 2020). Most artificial AMPs are prepared by chemical synthesis based on amino acid sequences. However, the high cost of raw materials, specialized equipment, low yield, and challenges in purification make chemical synthesis of AMPs costly (Lyu et al., 2023). Chemical synthesis of peptides generally includes liquid-phase synthesis, solid-phase synthesis, and peptide fragment synthesis. Solid-phase peptide synthesis (SPPS) is the most advanced and standard method for producing synthetic peptides (Collins et al., 2023). In particular, it is often necessary to synthesize longer and more complex peptides using SPPS. SPPS has advanced from laboratory-scale to industrial-scale production, enabling automation and large-scale manufacturing (Deo et al., 2022). Acyldepsipeptides (ADEPs) are cyclic AMPs produced by *Streptomyces hawaiianus*. In a recent study, Cobongela et al. (2023) synthesized an anionic ADEP analog by SPPS. The percentage yield of the synthesized peptide could be increased to more than 37% with a purity greater than 96%. However, SPPS still faces major challenges in its use, such as high cost, insufficient purity, and low efficiency.

Compared to natural peptides, synthetic peptides have a better antimicrobial effect and a broader antimicrobial spectrum and usually exert antimicrobial activity at low concentrations (Lima et al., 2021; Zhou et al., 2023). Notably, the antimicrobial mechanism of synthetic and natural peptides is the same, suggesting that they have similar properties.

## 3 Structure of AMPs

AMPs come from a wide range of sources, resulting in great structural diversity. AMPs are almost always positively charged with a small amount ranging from +1 ~ +9 (Chettri et al., 2024), crucial in exerting their antimicrobial, anticancer, and wound-healing-promoting effects. Most AMPs contain amphiphilic structures. The amphiphilic structure can make the AMPs conformation more flexible and is also regarded as the key structural factor influencing AMPs activity. Hydrophobic amino acid residues enhance the duration of AMPs interaction with the membrane and play a crucial role in their antibacterial activity. However, increasing hydrophobicity does not necessarily increase the antimicrobial activity of AMPs and may even increase hemolysis (Wang et al., 2021b) because increasing hydrophobicity may alter the AMPs secondary structure.

**Figure 1:**
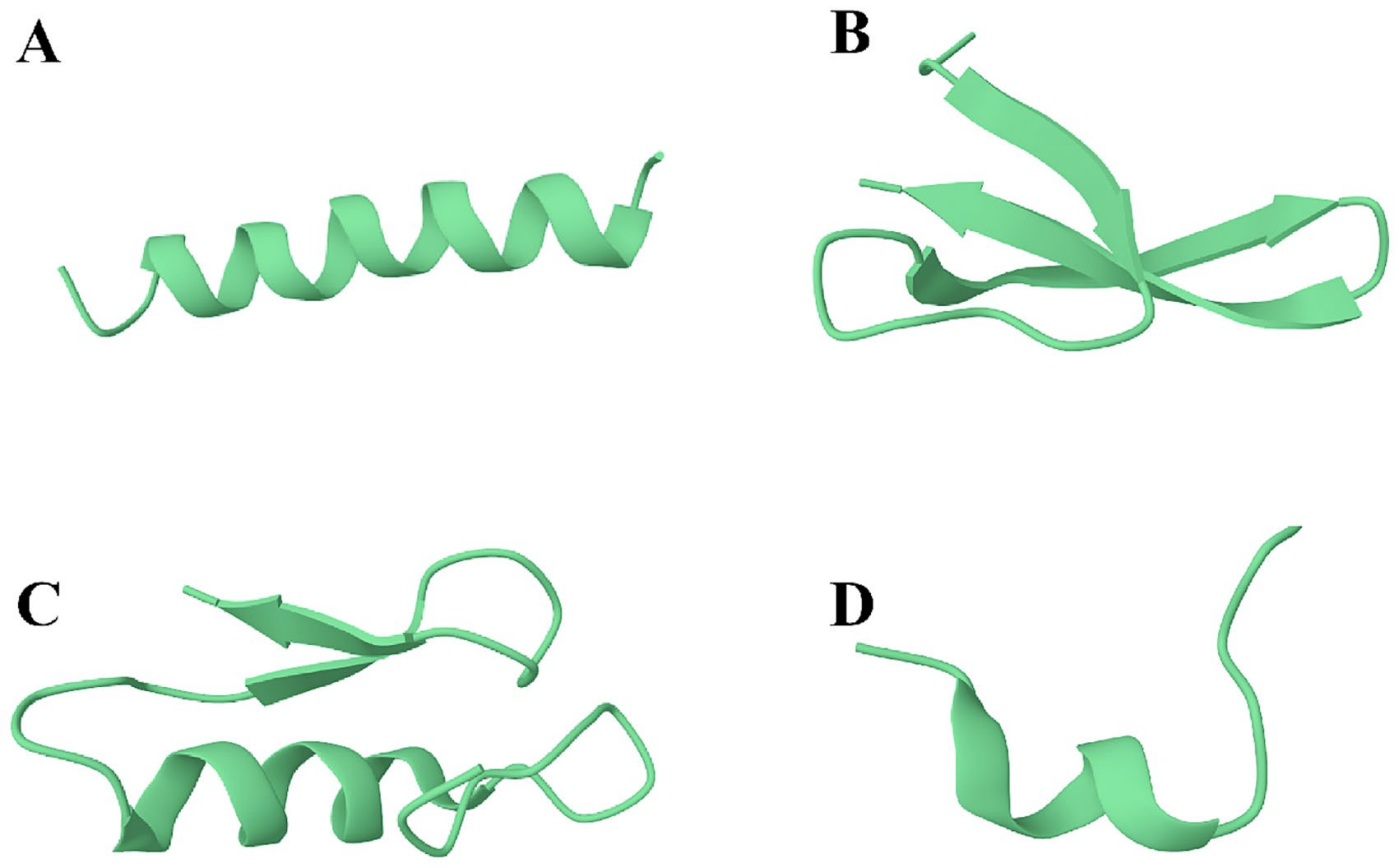
Examples of the four AMPs structural categories. **(A)** α-helical peptides, e.g., Magainin-2 from *Xenopus laevis* (PDB: 2MAG); **(B)** β-sheet peptides, e.g., Alpha-Defensin from *Macaca mulatta* (PDB: 2K1I); **(C)** αβ-peptides, e.g., MP1102 from *Pseudoplectania* (PDB: 6 K51); **(D)** non-αβ peptides, e.g., Tritrpticin from pig bone marrow (PDB: 1D6X).

The primary, secondary, and tertiary structures of AMPs are diverse, with its secondary structure being the main feature of AMPs and the primary basis for the current classification of AMPs (Piotto et al., 2012; Wu et al., 2018). Based on the secondary structure of AMPs, they can be classified into four major categories, namely, *α*-helical peptides, *β*-sheet peptides, αβ-peptides, and non-αβ peptides (Ji et al., 2024). Examples of the four AMPs structural categories are shown in Figure 1. As shown, Magainin-2 has a helically coiled forward shape (Figure 1A); the structure of Alpha-Defensin is composed of three almost fully extended polypeptide chains aggregated laterally (Figure 1B); the structure of Plectasin derivative (MP1102) consists of two extended polypeptides and one helical peptide (Figure 1C); the structure of Tritrpticin consists of one randomly curled peptide (Figure 1D). The sources, characteristics, and roles of the different structural types of AMPs are summarized as shown in Table 4. The magnitude of the number of structural AMPs in the four categories is *α*-helical peptides > αβ-peptides > β-sheet peptides > non-*α*β peptides (Ripperda et al., 2022). There is also a large portion of AMPs secondary structure that has not yet been determined (Jiang et al., 2021). In general, the properties of AMPs, such as charge, molecular weight, secondary structure, hydrophobicity, amphiphilicity, and the type and number of amino acid residues, are critical for their physicochemical properties and biological activities of the peptides.

**Table 4:** Summary of the four AMPs structural category sources, characteristics and roles.

Structure Type	Name	Source	Features	Effect	References
α-helical peptides	Alrn-6924	Structure modification synthesis	Good cell permeability, solubility, and favorable pharmacokinetic	Anti-tumor effect	Guerlavais et al. (2023)
	Synthetic peptides	Structure modification synthesis	9 lysine and 1 arginine residue、has a charge of +10	Both antimicrobial and anticancer activity	Thankappan et al. (2021)
	Dendrocin-ZM1	*Zataria multiflora* Boiss	With a net charge of +7 and 54% hydrophobicity	A remarkable antimicrobial candidate	Seyedjavadi et al. (2022)
β-sheet peptides	Collagencin peptide (GLPGPLGPAGPK)	Fully synthetic	Amphiphilic and cationic, with a lysine residue in the C-terminal region	Broad-spectrum antibacterial effect	Ennaas et al. (2016)
	MCNDCGA peptide (termed MOp3)	*Moringa oleifera* seeds	Hydrophobic anionic AMPs rich in β-sheet structures	Potent antimicrobial activity and negligible hemolytic activity	Wang et al. (2023c)
	Capitellacin	The marine polychaeta *Capitella teleta*	The β-sheet structure has a distinct right-handed	A promising broad-spectrum antibacterial agent	Panteleev et al. (2020)
	Arenicin-3	Lugworm	Consisting of 21 amino acids, cationic and amphiphilic	A broad spectrum of antimicrobial activity	Edwards et al. (2022)
αβ-peptides	Panusin	Hemocytes of the spiny lobster *Panulirus argus*	Contains one α-helix and three antiparallel β-sheets	A prospective anti-infective peptide lead	Bello-Madruga et al. (2023)
	Plectasin	The saprophytic ascomycete *Pseudoplectania nigrella*	Contains 40 amino acids, stabilized by three disulphide bonds	Promoted the growth and immunity of yellow-feathered chickens.	Zhang et al. (2021b)
	Myticofensins B	*Mytilus* coruscus	Contains one α-helix and two β-sheets structures	Broad-spectrum antimicrobial activity	Liu et al. (2022)
Non-αβ peptides	Kamp-10	Fully synthetic	Has a net charge of zero, no basic amino acids	Antibacterial effect	Lee et al. (2016)
	Indolicidin	Bovine neutrophils	13 amino acid cationic, with 38% of tryptophan and 28% of proline	Antibacterial and antiviral effects	Saeed et al. (2022)
	Tritrpticin	Pigs	13 amino acids with 4 arginine residues and 3 consecutive tryptophan residues	Antibacterial effect	Chan et al. (2006)

### 3.1 α-Helical peptides

α-helical peptides make up the largest and most studied group of AMPs, representing 30–50% of all AMPs with known secondary structures. α-helical peptides have been intensively studied in insects, fish, amphibians, mammals, and plants (Lachowicz et al., 2020). This class of AMPs represents the most potent and stable forms found in nature, efficiently facilitating binding to cellular membranes (Hernandez-Aristizabal and Ocampo-Ibanez, 2021; Thankappan et al., 2021). They are also more resistant to enzymatic, chemical, and thermal degradation, offering higher stability. They ensure structural stability mainly through electrostatic and hydrophobic interactions and hydrogen bonding.

The α-helical structure is vital for the antibacterial activity of the cationic host defense peptide family. α-helical peptides usually become linear or unstructured in water. However, they present an amphiphilic helical structure in bacterial cell membranes, which is a prerequisite for insertion into bacterial cell membranes (Qu et al., 2023). Classical *α*-helical peptides include LL-37, bee venom peptides, aspergillus, pexiganan, esculentin-2, magainins, and polybia-MP1 (Seo et al., 2012; Luong et al., 2020). e.g., magainins are typical members of the α-family. Magainin-2 is a 23 amino acid residue AMP obtained, which is unstructured in aqueous solvents, but has an α-helical structure in acidic phospholipid bilayers (Hasan et al., 2022). The amphiphilic structure and helicity of α-helical AMPs determine their activity; the higher the helicity, the better the activity, and the higher the specificity (Hassan et al., 2021; Hasan et al., 2022). They are often enriched in lysine, leucine, alanine, and glycine (Zou et al., 2020; Dini et al., 2022). For example, adepantin is rich in glycine and lysine, has high antibacterial activity, and has extremely low hemolytic activity. It is also highly selective against Gram-negative bacteria (Wang, 2023).

### 3.2 *β*-Sheet peptides

β-sheet structures are common in plants and help maintain the stability and activity of AMPs. This type of structures needs to contain at least two β-strands containing 2 ~ 10 cysteines residues and 1 ~ 5 disulfide bonds (Liu et al., 2021). Most β-sheet peptides contain cysteine residues, which form a disulfide bond between residues that are highly hydrophobic (Kumar et al., 2018; Zhou et al., 2023). The disulfide bond also improves resistance to protein hydrolytic degradation. Notably, when the disulfide bond is stabilized, the resulting linear polypeptide retains its antimicrobial properties even if the β-sheet structural peptide is disrupted. Small-sized β-sheet AMPs are highly resistant to proteolytic degradation. Common β-sheet peptides include β-defensins, drosocin, apidaecin, thanatin, and rattusin. β-sheet peptides are usually enriched in leucine, alanine, glycine, cysteine, and lysine (Bin Hafeez et al., 2021).

### 3.3 *α*β-peptides

AMPs can contain both α-helical and β-sheet structures, which are known as αβ-peptides. αβ-peptides similarly stabilize molecules by disulfide bonds, and the structures are tryptophan-rich and proline-rich. The advantage of this type of AMPs is that it can strongly target and disrupt bacterial cell membranes (Bin Hafeez et al., 2021). Less research has been done on αβ-peptides, and the classic peptides of this class are plant defensins and insect defensins. With their stable structure and ease of application for peptide engineering, insect defensins have been considered a promising candidate for developing novel antibiotic lead molecules (Robles-Fort et al., 2021). Insect defensins contain six cysteines connected by two disulfide bonds to the C-terminal β-sheet and α-helical structures. Interestingly, plant defensins with this type of structure are mainly inhibitory to fungi, whereas animal defensins are mainly inhibitory to bacteria (Sher Khan et al., 2019; Yan et al., 2022).

### 3.4 Non-αβ peptides

Non-αβ structures refer to certain AMPs that do not contain an αβ structure, also known as elongated peptides, which do not have a regular secondary structure. Non-αβ structures are not formed by hydrogen bonding between amino acid residues, making it difficult to form spatial secondary structures. These AMPs have a unique antimicrobial mechanism because they do not contain αβ structures. They can bind to molecules involved in bacterial growth and inhibit their function, ultimately leading to bacterial death. These AMPs are usually rich in one amino acid, such as arginine, tryptophan, proline, or cystine residues (Yasir et al., 2018; Kumar et al., 2024). Indolicidin is a classical non-αβ peptide. Indolicidin is derived from bovine neutrophils and contains 13 amino acid residues, 5 tryptophan, and almost half are hydrophobic. Indolicidin has diverse biological targets and activities, offering significant potential for applications across various fields (Batista Araujo et al., 2022). These peptides exhibit sensitivity to extracellular proteases produced by Gram-positive bacteria, resulting in more potent activity against Gram-negative bacteria (Liu et al., 2021). In addition, Lee et al. (2016) identified the first human AMP (KAMPs-19) with a non-αβ structure. KAMPs-19, derived from human epithelial keratin 6A, is glycine-rich, contains a novel amphiphilic structure, and possesses bactericidal and cytoprotective activity.

## 4 Optimization strategies of AMPs

The structure of AMPs has an important influence on their physicochemical properties and biological activity. Modification of their structure can achieve the optimization of AMPs. Most AMPs have various applications due to their excellent water solubility, thermal stability, low immunogenicity, low susceptibility to drug resistance, and broad-spectrum antimicrobial properties. However, many natural AMPs face more confusion in their applications, such as high development cost, short half-life, susceptibility to degradation by proteolytic enzymes, low bioavailability, sensitivity to environmental factors, higher hemolysis, and toxicity (Sarkar et al., 2021). Therefore, we need to optimize the AMPs to meet the demands of commercial applications. The current optimization strategies for AMPs mainly include improving the activity and stability of AMPs and the dosage form.

### 4.1 Increased activity

Most AMPs have weak antimicrobial effects compared with antibiotics. Researchers have made extensive explorations to improve their activity further. Chemical modification technology is an important means of AMPs optimization. The modifications used to enhance the activity of AMPs often include amino acid substitution, halogenation, amidation, cyclization, and other methods.

Typically, only a few essential amino acids in AMPs are required for antimicrobial activity. We can modify the residues of AMPs to improve their activity. The hydrophobicity of AMPs plays a crucial role in determining their partitioning into the membrane lipid bilayer, a key factor for membrane permeabilization (Deshayes et al., 2022). In a recent study, Yang et al. (2024b) synthesized a series of novel Brevicidine derivatives (Brevicidine 22) by doping Brevicidine with different hydrophobic amino acids, N-terminal fatty acids, and different types and amounts of positively charged amino acids. Brevicidine 22 exhibited strong biofilm inhibition, eradication, and rapid bactericidal effects. In particular, the stability of Brevicidine was enhanced, its antimicrobial spectrum expanded, antimicrobial potency increased, and bacterial resistance was reduced. In addition, amidation is a simple way to enhance the stability and activity of AMPs *in vivo*. For example, acetylation enhances the bacteriostatic efficacy of L163 through dual mechanisms: promoting membrane bilayer destabilization through structural disruption and facilitating DNA-binding interactions (Li et al., 2022). Chowdhury et al. (2020) also modified a novel AMP (QAK) by acetylation. It was shown that acetylated QAK increased antimicrobial activity by disrupting the bacterial cell membrane and releasing more intracellular substance *β*-galactosidase than unacetylated QAK.

**Figure 2:**
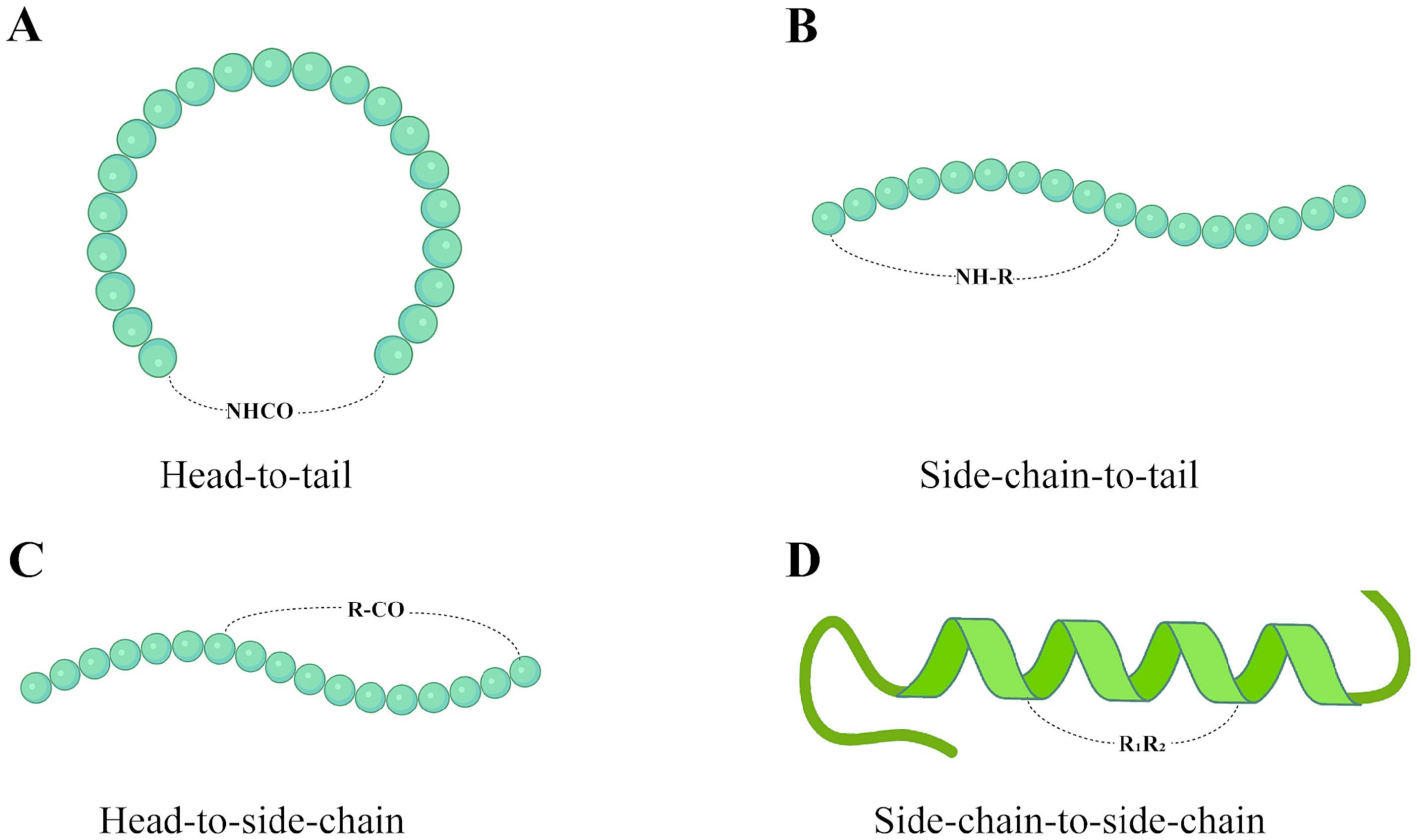
Four AMP cyclization technologies: **(A)** Head-to-tail. **(B)** Side chain-to-tail. **(C)** Head-to-side chain. **(D)** Side chain-to-side chain. The “R” group represents the side chain group of the amino acid. Created with BioRender.com

Cyclization is a commonly used method to enhance AMPs activity and can be performed by cyclizing the peptide chain through disulfide or amide bonds (Han et al., 2021). There are currently four classical peptide cyclization methods: head-to-tail, side chain-to-tail, head-to-side chain, and side chain-to-side chain. The four types of cyclization of peptide drugs are shown in Figure 2. Head-to-tail technology removes the N-terminal amine and C-terminal acid groups to form an amide bond, thereby reducing the polarity of the compound. Reducing the polarity of the compound increases its cell membrane permeability, thereby improving its stability to proteases, increasing its bioavailability and enhancing its efficacy (Tivari et al., 2022). Etayash et al. (2020) reported three synthetic strategies for cyclizing linear AMP (IDR-1018). These were head-to-tail cyclization [C1 strategy (Figure 2A)], side chain-to-tail cyclization [C2 strategy (Figure 2B)], and disulfide bond cross-linking [C3 strategy (Figure 2D)]. It was found that all three synthesized ring derivatives showed enhanced protein hydrolytic stability and reduced aggregation. In particular, the *in vivo* antimicrobial activity of IDR-1018 derivative C2 was enhanced. This enhanced in vivo efficacy is likely due to increased stability resulting from the conformational rigidity of the peptide, as well as other alterations in physicochemical properties. In addition, Teng et al. (2016) synthesized cyclic *γ*-AA2 by cyclization of γ-AApeptides via head-to-side chain cross-linking strategies (Figure 2B). Cyclization of γ-AApeptides can enhance the rigidity of the sequence, which significantly improves its activity. There are fewer studies on halogenation reactions to enhance the activity of AMPs. However, halogenation is a valuable strategy for modulating the properties of bioactive molecules. Jia et al. (2019) modified Jelleine-I by halogenation. They found that the *in vitro* antimicrobial activity, anti-biofilm activity, and in vivo antimicrobial effect of the halogenated derivatives were increased by 1 ~ 8 times.

Synergistic combinations of some AMPs with conventional antibiotics demonstrate potentiated efficacy against both drug-sensitive and multidrug-resistant bacterial strains (Zhu et al., 2022). Future research efforts in this direction can be increased, reducing the amount and cost of AMPs, facilitating synergistic effects and enhancing antibacterial effectiveness.

### 4.2 Improved stability

Various proteolytic enzymes easily target peptides, and thus they are poorly stabilized in vivo. The stability of peptides against proteases has always been a major requirement for developing peptide therapeutics. However, AMPs face multiple stability challenges in practical applications, such as poor protease stability, serum stability, and metabolic stability, which limit their widespread clinical use. To improve the stability of AMPs, researchers have optimized them mainly by modifying the amino acid sequence. AMPs cyclization, glycosylation, lipidation, polyethylene glycolization, N-methylation, N-terminal acetylation, and C-terminal amidation have been suggested as effective methods for improving stability. Table 5 compares the effects of the above improved methods on the physical and chemical properties and effects of AMPs. Appropriate improved methods can be adopted according to research needs.

**Table 5:** Effects of different improvement methods on the physicochemical properties and effects of AMPs.

Name	Improved methodology	Changes in physical and chemical properties	Effect	References
KR-12	Main chain cyclization and dimerization	Increased net surface charge	Enhanced antimicrobial activity and protein hydrolysis stability	Gunasekera et al. (2020)
L163	N-terminal acetylation	Reduced net surface charge	Enhanced stability to pH, plasma, and trypsin degradation	Li et al. (2021a)
LL-III	Glycosylation	-	Increased resistance of peptides to protease action	Tortorella et al. (2023)
Amphiphilic K4F4 peptide	Lipidation	Reduced water solubility	Improved serum stability and antimicrobial activity	Mendes et al. (2024)
Anoplin	N-methylation	Reduced hydrophobicity and α-helix content	10^4^ times increase in resistance to trypsin degradation	Liu et al. (2020)
Pexiganan	Complexation with macrocyclic compounds	-	Reduced hemolytic toxicity and enhanced protein hydrolytic stability	Chen et al. (2021a)

“-” indicates that this information is not mentioned in the literature.

To improve the stability of Lasioglossin-III (LL-III) and prevent protease degradation, Tortorella et al. (2023) synthesized glycosylated LL-III (g-LL-III) by covalently linking N-acetylglucosamine (NAG) to the Asn residue in the N-terminal region of LL-III. The results showed that g-LL-III effectively increased the stability of protease degradation. Moreira Brito et al. (2022) synthesized an AMP (LyeTx I-b) using total synthesis. They coupled it to polyethylene glycol (PEG) to synthesize LyeTx I-bPEG. Enzymatic degradation experiments revealed that LyeTx I-bPEG remained highly stable after 24 h of exposure to trypsin or proteinase K, whereas LyeTx I-b was completely degraded by either enzyme within 6 h. Some studies have coupled N-methylated and fatty acid Anoplin to improve its limitations. The results indicated that N-methylated lipopeptides were 10^4^ to 10^6^ times more stable against degradation by trypsin and pancreatic coagulation protease than natural Anoplin (Liu et al., 2020). Acetylation can block the degradation sites of proteases and AMPs, thereby enhancing the stability of AMPs and prolonging their effective time in plasma (Li et al., 2022). For example, Kuzmin et al. (2017) modified the *β*-hairpin tachyplesin I by N-terminal acetylation and C-terminal amidation. The modified peptide demonstrated significantly improved stability in human serum, which also increased both specific and non-specific cytotoxicity of tachyplesin I. Peptide cyclization is also a promising approach to enhance their stability. Cyclization slows down protease cleavage by tightening amino acid side chains.

New strategies have also been developed to enhance the stability of AMPs. Zhu et al. (2020) created a series of novel AMPs with high stability based on protease-specific cleavage sites and symmetric end-labeling. Among them, II-I4-II demonstrated high stability against various proteases, saline, serum, and under acidic, alkaline, and thermal conditions. Recently, Guo et al. (2024a) also reported a new modification strategy involving the introduction of fluorinated sulfonyl-*γ*-AA into the protease-sensitive site of Feleucin-K3. Using this approach, they synthesized a series of novel Feleucin-K3 derivatives containing fluorinated sulfonyl-γ-AA. The results showed that this new approach effectively improved the stability and activity of Feleucin-K3. Among them, CF3-K11 was the best peptide, which has strong therapeutic potential with a faster bactericidal effect.

The combination of AMPs and nanomaterials can also enhance the stability of AMPs while maintaining their bactericidal activity. Li et al. (2017) used β-cyclodextrin to form an inclusion complex with CM4. Under the protection of β-cyclodextrin, the antibacterial activity of CM4 is not affected, and the storage stability of CM4 and its stability to proteinase K, trypsin and pepsin *in vivo* and *in vitro* are improved. Similarly, studies have used nanogels and microneedle patches to encapsulate AMPs, which improved the stability and biocompatibility of AMPs (Wang et al., 2023).

### 4.3 Improvement of dosage forms

More AMPs administered in vivo are subject to hemolysis and poor stability, while topical administration has a low ability to penetrate the bloodstream. Therefore, AMPs are often administered locally or by injection. Topical administration is currently the most practical method for delivering AMPs, which provides higher peptide concentrations at the site of action and reduces adverse effects. To date, the FDA-approved delivery modes for AMPs for infected skin indications have all been in the topical phase. Developing in vivo delivery systems for AMPs is critical to improving the mode of delivery, expanding the range of applications, and achieving subsequent clinical applications. Delivery carrier materials not only help to improve the bioavailability, biocompatibility, solubility, prolong the half-life, overcome hemolysis, reduce cytotoxicity, improve stability and efficacy of AMPs, but also to obtain targeting and achieve the effect of controlled release of the drug (Martin-Serrano et al., 2019).

Recently, carrier materials for AMPs have gradually become the research focus. Currently, novel dosage forms of AMPs mainly include nanomicelles, liposomes, hydrogels, creams, dendritic macromolecules, and metal nanoparticles (Wang et al., 2021a). Table 6 compares the effects of the above different dosage forms on the efficacy of AMPs, which can be selected according to the needs of the study. Jeong et al. (2023) developed an injectable supramolecular hydrogel, and then loaded AMPs into the hydrogel. It was found that the AMPs hydrogel had greater stability compared to natural AMPs and greatly improved chronic wound healing in diabetes. Currently, the most researched systems regarding the delivery system of AMPs are metal nanoparticle formulations, especially gold nanoparticles and silver nanoparticles (AgNP). AgNP also possesses excellent antimicrobial properties, especially when used synergistically with other antimicrobial agents (Fadaka et al., 2021). For example, Xu et al. (2021) used polydopamine (PDA) as a green reducing agent and binder and modified AgNPs with AMP to develop an AMP@PDA@AgNP nanocomposite. It was shown that AMP and silver nanoparticles could synergistically affect the treatment of bacterial infections. The AMP@PDA@AgNP nanocomposite could disrupt the bacterial biofilm by inhibiting the expression of biofilm-related genes.

**Table 6:** Effect of different drug carriers on AMPs.

Dosage form	Material	Name	Effect	References
Nano micelles	PEG-PCL	Protonectin	Enhanced the solubility of protonectin	Eskandari et al. (2021)
Hydrogel	O-CMCS/SAP	Mel-d1	Non-toxic and non-hemolytic, with sustained release capability, speed up wound healing	Huan et al. (2022)
Nanoparticles	Chitosan	Octominin	Showed reduced toxicity and enhanced antibacterial activity against *Candida albicans* and *Acinetobacter baumannii*	Jayathilaka et al. (2022)
Nanoparticles	Calcium phosphate	LL-37	Protected LL-37 from degradation by proteinase K after 4 h of incubation	Tsikourkitoudi et al. (2020)
Silver nanoparticles	AgNO_3_	Cecropins	Showed significantly higher stability and activity, with a lower risk of developing resistance	Muchintala et al. (2020)
Silver nanoparticles	AgNPs	(LLRR)_3_	Improved performance of nanomaterials and reduced hemolytic activity and cytotoxicity of AMP	Li et al. (2024b)
Microspheres	Poly (lactic-co-glycolic acid)	OH-CATH30	Improved bioavailability and powerful antimicrobial efficacy	Jiao et al. (2023)
Microcapsules	Hypromellose phthalate	Ctx(Ile^21^)-Ha	Reduced the mortality rate in laying hens caused by resistant *Salmonella enteritidis*	Roque-Borda et al. (2021)
Polyion complex nano-objects	Poly(ethylene oxide)-poly(acrylic acid)	P6	Enhanced anti-tumor activity of p6	Răileanu et al. (2022)
Gold nanoparticles	Gold chloride	VG16KRKP	Targeted VG16KRKP to fight *Salmonella Typhi* effectively	Chowdhury et al. (2017)

Altering the AMPs delivery system can effectively improve the hemolytic and low toxicity of AMPs. For example, Almaaytah et al. (2017) used chitosan nanoparticles (CS-NP) as a novel in-house designed delivery system for effective ultrashort AMP (RBRBR). The study found that the highest concentration of the RBRBR-CS-NP formulation exhibited a hemolytic percentage of 3.6%, compared to 13.1% for the free peptide. This result indicates that CS-NP significantly reduces the hemolytic activity and toxicity of the free peptide on mammalian erythrocytes. Liposomes are a common delivery system for AMPs, with their size, lipid composition, and surface modifications easily adjustable to modify the physicochemical properties of AMPs (Drayton et al., 2020). In a recent study, Camila Herrera et al. (2024) encapsulated thuricin CD into anionic liposomes. It was found that the antimicrobial activity, gastrointestinal fluid stability, and storage stability of the peptide at room temperature were improved compared to free thuricin CD.

In addition to nanomaterials acting as a carrier system for AMPs, the combination of the two may also result in synergistic effects, enabling synergistic multi-mechanism sterilization as well as slowing down the evolution of multidrug-resistant bacteria. In the future, artificial intelligence can predict the best combination of AMPs and nanomaterials and optimize the structure of AMPs and nanomaterials to enhance their efficacy. In addition, some nanomaterials can be targeted to sites of infection and tumors, thereby reducing the systemic toxicity of AMPs (Hussain et al., 2018; Ojha et al., 2022). Currently, there are few studies on the development of delivery systems for AMP-targeted therapy. In the future, research in this direction could be strengthened for precise and targeted delivery of AMPs to their site of action. *In vivo* pharmacodynamic and toxicological studies of nano-AMPs complexes can also be enhanced to promote their entry into clinical trials. However, the application of the above AMPs delivery systems may pose risks in terms of biocompatibility, immunogenicity and *in vivo* metabolism. Therefore, not only is there a need to optimize the design of nanomaterials to minimize side effects, but safety data from long-term treatment or repeated dosing is also needed to avoid potential risks. In addition, the synthesis process of nanomaterials can be complex and costly, making it difficult to achieve large-scale production. Green synthesis methods can be explored to reduce the cost of preparing nanomaterials.

Overall, in addition to optimized methods such as chemical modification, nanocarrier delivery, and co-administration with antibiotics, genetic engineering can be used to produce AMPs on a large scale. In recent years, the global market for AMPs has continued to grow steadily. In 2022, the market size of AMPs is around $4.6 billion (Mihooliya and Kumari, 2024), which shows a promising prospect. The above optimization methods can accelerate the commercial application process of AMPs, but there are also some disadvantages on the road to commercialization. Table 7 summarizes the impact of AMPs optimization methods on efficacy and commercial application.

**Table 7:** The impact of AMPs optimization methods on efficacy and commercial application.

Optimization Methods	Efficacy	Commercial viability		References
		Advantages	Disadvantages	
Chemical modification	Improve biological activity and stability; reduce toxicity	Mature modification technology	High cost	Lai et al. (2022)
Nanocarrier delivery	Improve solubility, stability, bioavailability and targeting	Improve the delivery method of AMPs and enhance market competitiveness	High technical threshold, high R&D cost, and high commercialization supervision requirements	Liu et al. (2019)
Combination with antibiotics	Improve antimicrobial activity and reduce the dosage of a single drug	Expanding the target patient group, high market demand	May increase side effects, require more clinical trials, and extend the commercialization cycle	Gonçalves et al. (2025)
Genetic Engineering	Highly active and stable AMPs can be designed	Reduce costs and achieve large-scale production	The technical threshold is high; the produced AMPs may be contaminated by endotoxins, affecting product quality	Deo et al. (2022)

## 5 Main activities of AMPs

In addition to optimizing AMPs, exploring and clarifying the pharmacological activities and effects of AMPs is also an important means to expand their application. In addition to AMPs potent antibacterial activity, other well-studied activities are immunomodulatory, antitumor, and antiviral, which have stimulated continued interest in such molecules. The exact mechanisms underlying the various activities of AMPs are not yet fully understood. Below is an overview of the main activities of AMPs and the proposed mechanisms of action.

### 5.1 Antimicrobial activity

Antimicrobial activity is AMPs most important and prominent activity. The presence of components such as lipopolysaccharides and lipophosphatidic acid on the bacterial surface makes the bacterial surface carry a negative charge. Therefore, cationicity is a key property of the antimicrobial action of AMPs. AMPs have a broad spectrum of inhibitory and antimicrobial properties, and can be used in various applications, such as in treating bacteria. In addition to their bactericidal and bacteriostatic properties, AMPs disrupt the adhesion and subsequent colonization of pathogenic bacteria (Lyu et al., 2023).

AMPs have great potential to address the problem of antibiotic resistance. In a recent study, Suchi et al. (2023) isolated a novel AMP (YS12) from *bacillus velezensis* CBSYS12. Their antimicrobial assay showed that YS12 possessed strong antibacterial activity against Gram-positive and Gram-negative bacteria, with minimum inhibitory concentration (MIC) values ranging from 6 ~ 12 μg/mL. In particular, YS12 exhibited stronger biofilm eradication activity against drug-resistant bacteria than commercial antibiotics, suggesting it could prevent antibiotic resistance. Tian et al. (2024) synthesized a novel AMP (MR-22) using total synthesis. MR-22 was found to have good resistance against multidrug-resistant *E. coli* and inhibited *E. coli* in a dose-dependent manner with MIC and minimum bactericidal concentration ranging from 4 ~ 32 μg/mL.

**Figure 3:**
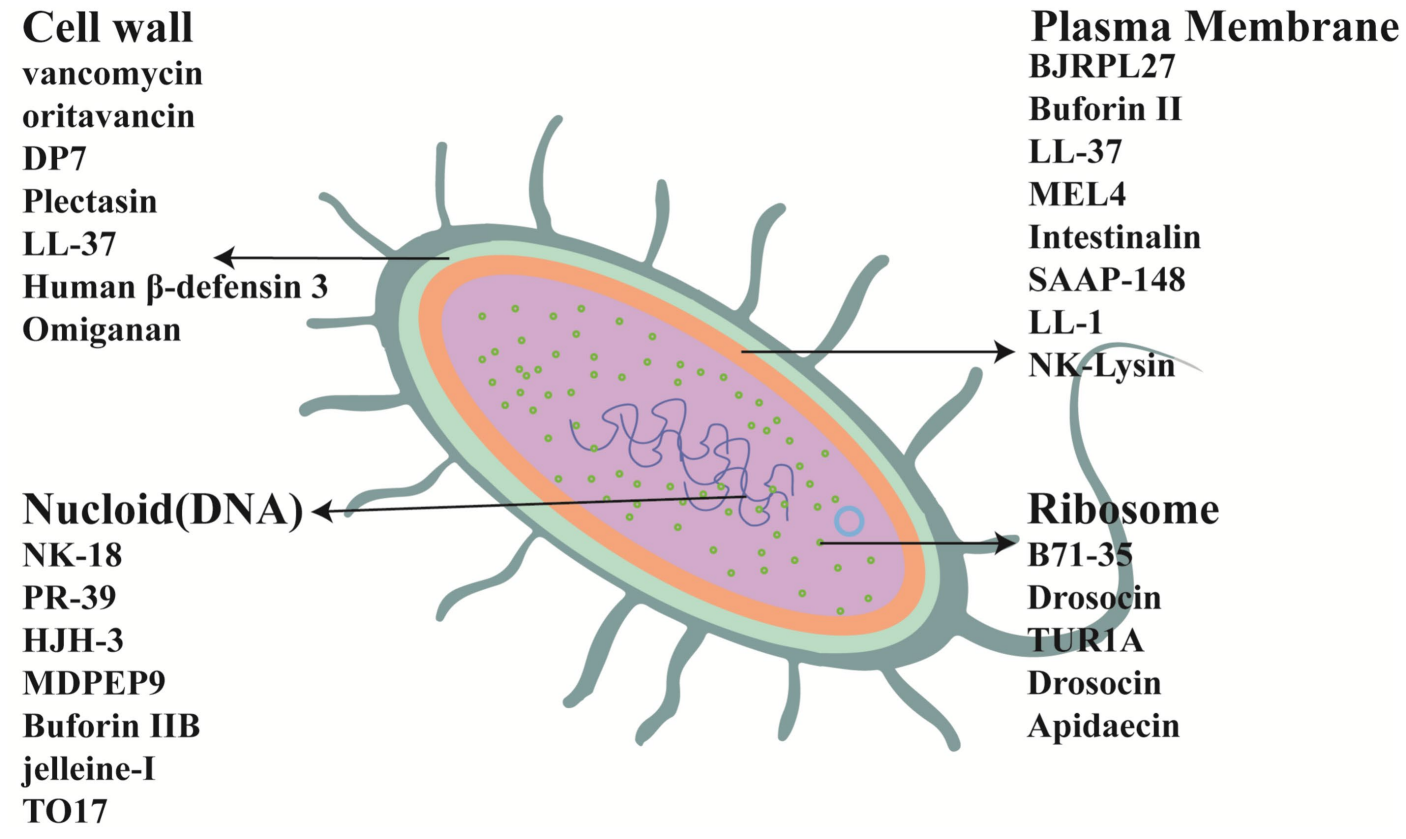
Examples of targets for AMPs.

The antibacterial mechanisms of AMPs are very diverse and have not yet been fully elucidated. In general, the antibacterial mechanisms can be divided into two categories: membrane-disrupting AMPs and non-membrane-disrupting AMPs (Masimen et al., 2022). The targets of some AMPs are shown in Figure 3. In Figure 3 cell membrane targets, AMPs such as BJRPL27 can destroy the bacterial cell membrane, causing the cell membrane to depolarize, membrane permeability to change, intracellular macromolecules to leak, and ultimately leading to bacterial death (Nayab et al., 2022). In Figure 3 cell wall targets, AMPs such as Vancomycin can hinder the formation of bacterial cell walls, cell growth, and cause cell wall perforation and death. In Figure 3 nucloid targets, AMPs such as NK-18 can inhibit DNA replication to exert bactericidal effects (Bin Hafeez et al., 2021). In Figure 3 ribosome targets, AMPs such as Bac71-35 can bind to bacterial ribosomal proteins and inhibit protein synthesis (Mardirossian et al., 2014). Microbial cell membranes are a key target for most AMPs. For example, Buforin IIb has a unique mechanism that enables it to quickly cross the bacterial membrane without causing cell lysis, killing bacteria by interacting with intracellular macromolecules (Al-Benna et al., 2011). In another study, Wang et al. (2022) synthesized an antimicrobial lipopeptide (LP21) using total synthesis. LP21 possesses efficient antimicrobial activity, serum stability, and low cytotoxicity. Transmission electron microscopy and scanning electron microscopy were used to observe the membrane integrity of LP21 after its action on *E. coli* ATCC 25922. It was found that compared with the control group, the bacterial membrane of *E. coli* in the LP21 group exhibited wrinkles, small vesicles, and even rupture. Additionally, some cell contents were observed to flow out, proving that LP21 exerted its antimicrobial effect by disrupting the cell membrane. “Current research has not yet fully elucidated the molecular mechanisms underlying the antimicrobial activity of non-membrane-targeting AMPs. Studies have shown that non-membrane-disrupting AMPs can exert antibacterial effects through immunomodulation, cell wall destruction, inhibition of protein synthesis, and neutralization of endotoxins (Jiang et al., 2021; Datta et al., 2024). For example, Mardirossian et al. (2014) reported a proline-rich AMP that can specifically inhibit bacterial protein synthesis *in vitro* and *in vivo*.

### 5.2 Immunomodulatory activity

AMPs are also known as host defense peptides because of their immunomodulatory benefits. They have been described as potent modulators of inflammation and neutralizers of toxins (Deshayes et al., 2022). The critical role of AMPs in host natural immunity is not limited to killing pathogenic microorganisms that invade the human body, but also shows a variety of powerful effects at different stages of the host’s natural immune response.

AMPs can recruit chemokines, activate macrophages, promote T-lymphocyte transformation, and increase immunoglobulin secretion (Niyonsaba et al., 2009). If innate immunity cannot eliminate the infection, AMPs act as a signaling bridge between innate and specific immunity through signaling pathways that initiate and amplify host-specific immune responses. For example, LL-37 can bridge innate and acquired immunity by recruiting immune cells to infected sites and stimulating or modulating the acquired immune system by explicitly activating receptors on immune cells (Yang et al., 2020). Lymphocytes are critical immune cells in the organism, and their proliferation and differentiation are essential stages in the organism’s immune response process. Therefore, the immune level of the animal organism can be indirectly proved by detecting lymphocytes. Studies have shown that AMPs can directly interact with B and T lymphocytes to regulate the host’s acquired immune function (Lyu et al., 2023).

**Figure 4:**
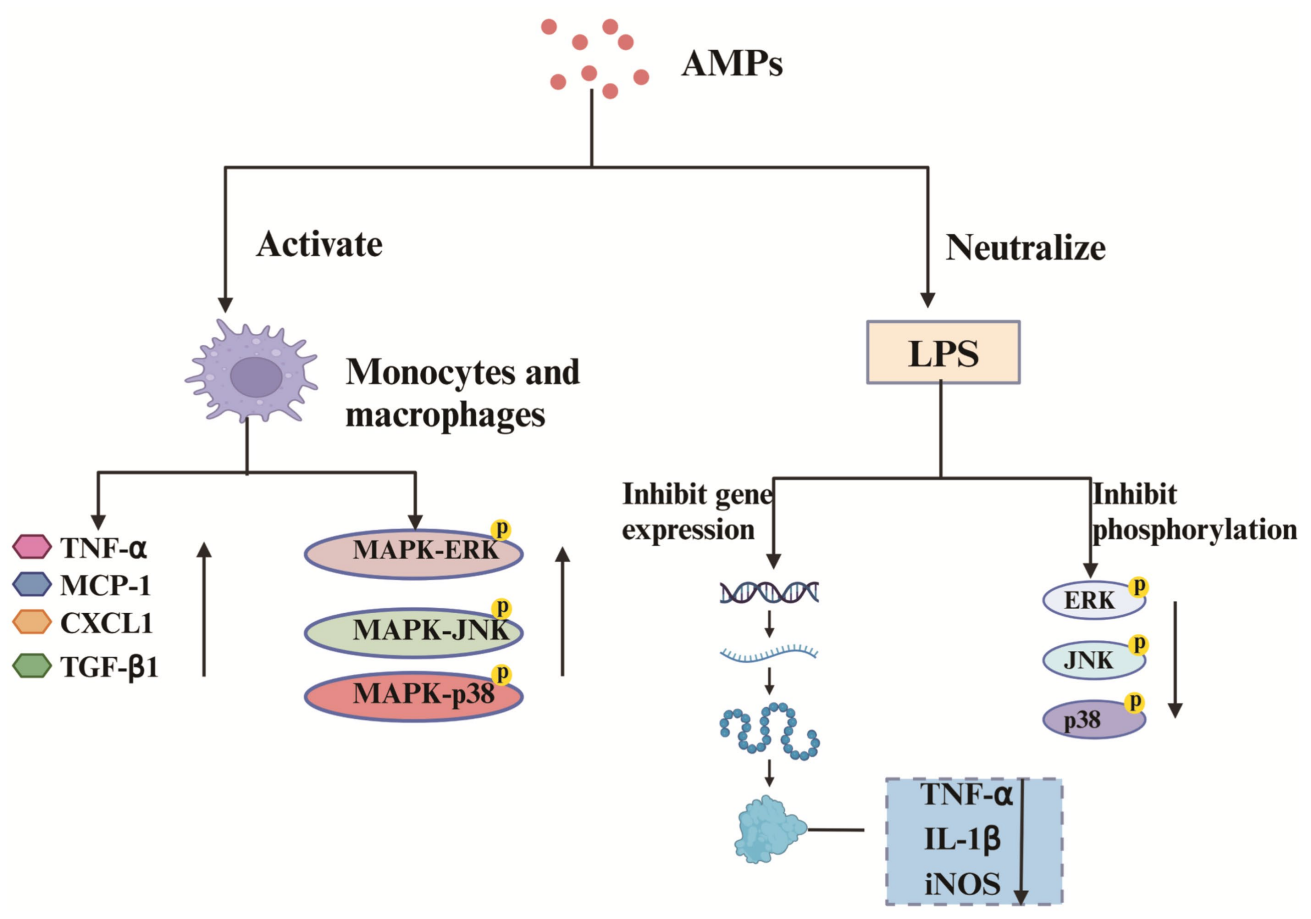
Regulatory mechanisms of TK-CATH on macrophages and inhibition of pro-inflammatory cytokine gene expression and protein production in macrophages induced by LPS. Created with BioRender.com

LL-37, polymyxins, teicoplanins, cathelicidins, and bacitracin are important immunomodulatory peptides. AMPs enhance the intracellular bactericidal effects of neutrophils and macrophages, strengthening the host immune system. Luo et al. (2021) were the first to isolate from the skin of the salamander *Tylototriton kweichowensis*, a novel net charge of −3 anionic cathelicidin (TK-CATH). It was found that TK-CATH had no direct antibacterial activity but showed potent anti-inflammatory and wound healing-promoting activities. TK-CATH enhances macrophage proliferation and activation in injured mice in vivo and promotes the production of TNF-*α*, MCP-1, CXCL-1 and TGF-β1 by macrophages. It also activates mitogen-activated protein kinase (MAPK) signaling pathways and increases the phosphorylation of phosphorylation of ERK, JNK and p38. In addition, TK-CATH neutralizes LPS toxicity and inhibits LPS-induced TNF-α, IL-1β and iNOS gene expression. It also inhibited LPS-activated inflammatory signaling pathways and reduced the phosphorylation of ERK, JNK and p38. The regulatory mechanism of TK-CATH on injured mouse macrophages and the mechanism of inhibition of LPS-induced pro-inflammatory cytokine gene expression and protein production in macrophages are plotted in Figure 4. In a recent study, He et al. (2024) synthesized a novel anionic AMP (Gy-CATH) with a net charge of −4. It was found that Gy-CATH had no direct antimicrobial activity, but showed significant preventive and therapeutic abilities against mice infected with *Staphylococcus aureus*, *Escherichia coli*, and *methicillin-resistant Staphylococcus aureus*. This is mainly because Gy-CATH exhibits potent immunomodulatory activity, enhancing macrophage-and neutrophil-mediated bactericidal functions through oxygen-dependent and oxygen-independent mechanisms. AMPs can also inhibit the expression of inflammatory factors, greatly reducing the inflammatory response and slowing down inflammatory damage. For example, Silva et al. (2016) synthesized a new synthetic AMP (clavanin-MO), which kills bacteria directly and has potent immunomodulatory activity. It can reduce the levels of pro-inflammatory cytokines IL-12 and TNF-α, helping to control excessive inflammatory responses.

### 5.3 Anti-tumor activity

Cancer patients undergoing long-term chemotherapy not only become resistant to conventional cancer treatments, but also become susceptible to pathogenic infections (Zhang et al., 2021a). AMPs are the only class of compounds that can effectively fight against a wide range of mixed microbial infections as well as cancer (Dong et al., 2024). In recent years, the antitumor activity of AMPs has become a hot research topic. The more studied AMPs with antitumor activity are defensins, Magainin-2, Cecropins, and others.

The anti-tumor mechanism of AMPs is similar to its antibacterial mechanism and is more complex. Its main anti-tumor mechanism has three aspects: 1. Direct contact with tumor cells and damage to cell membranes. 2. Inhibit the synthesis and proliferation of tumor cell DNA and damage to mitochondria, thereby inhibiting or killing tumor cells. For example, Epinecidin-1 from the ocean can induce cell death of non-small cell lung cancer by damaging mitochondria, increasing the level of reactive oxygen and disrupting redox balance (Yu et al., 2023). 3. Improve the body’s immunity, thereby identifying and killing tumor cells. Most studies have shown that AMPs exert their anticancer effects by disrupting cell membranes. This is largely because most AMPs are positively charged and amphiphilic, while most tumor cells carry a net negative charge on the cell membrane. This facilitates the selective binding of AMPs to the negatively charged cell membranes of tumor cells, forming transmembrane channels that disrupt membrane integrity and kill cancer cells (Dong et al., 2024). For example, He et al. (2020) isolated a novel α-helical AMP (LHH1) from *Lactobacillus casei* HZ1. It was found that LHH1 could dose-dependently inhibit the viability of C666-1, MGC803 and HCT116 cells. Whether LHH1 affected cancer cell membranes was observed by a confocal laser scanning microscope (CLSM). The results showed that the cell membranes of the three cancer cells were blurred, indicating that LHH1 could play an anticancer role by destroying the cell membranes of the three cancer cells. Mohammadi et al. (2023) found that moronecidin-like peptide (MLP) combined with anti-PD-1 treatment of melanoma enhances the anti-tumor immune response, thereby controlling tumor growth and increasing animal survival. Crucially, AMPs also showed significant activity against multidrug-resistant cancer cell lines, potentially replacing certain radiotherapy drugs to treat advanced stages.

Some AMPs are cytotoxic to many cancer cells, such as Brevenin-2R, temporin-1CEa, and Buforin IIb. Buforin IIb is a synthetic analog of buforin II, which is selectively cytotoxic to cancer cells such as leukemia, hepatocellular carcinoma, cervical carcinoma, prostate carcinoma, and colon carcinoma (Tornesello et al., 2020). Recently, Selvarathinam et al. (2023) found that the novel AMP (SKACP003) they identified has a unique mechanism of action against breast cancer. SKACP003 can target *β*-catenin and inhibit the Wnt signaling pathway. Dysregulation of Wnt signaling can inhibit the progression of most cancers.

AMPs offer notable advantages over conventional antitumor drugs, including higher specificity and the ability to overcome drug resistance. Currently, it is not possible to predict the anti-tumor effects of AMPs based solely on their structure. Future research in this direction could be strengthened to develop anti-tumor AMPs faster and more targeted.

### 5.4 Antiviral activity

Viral infections are at the root of many infectious diseases and can seriously threaten human and livestock life. Antiviral drugs are few and difficult to develop, and more antiviral drugs need to be designed to address these problems. Some AMPs are functionally diverse broad-spectrum antiviral molecules with equally complex mechanisms of antiviral action. Some more prominent antiviral AMPs include defensin, LL-37, tensin, cyclic peptide, and lactoferrin. LL-37 has been reported to inhibit viruses such as human immunodeficiency virus, influenza A virus, rhinovirus, cowpox virus, herpes simplex virus, Zika virus (ZIKV), and hepatitis C virus (Ahmed et al., 2019). A recent study has shown that exosome-loaded LL-37 also significantly inhibited ZIKV infection. Its mechanism of action involves directly inactivating viral particles, reducing host cell susceptibility, and interfering with viral replication (Wang et al., 2024a).

There are three mechanisms of action of AMPs antiviral mainstreaming. The first is the disruption of the viral envelope. For example, palicourein inhibits enveloped viruses (HIV) at a half-effective concentration of only 100 nM (Gustafson et al., 2004). Meng et al. (2024) found that Sparamosin_26-54_ exhibited potent antiviral activity against enveloped viruses, including WSSV, AngHV, and RGV. The mechanism of action is that it can disrupt viral envelopes through lipid-binding-mediated virus lysis. Sparamosin_26-54_ also reduces viral load in tissues, improves animal survival or reduces infection rates, and is used in aquaculture to combat viral infections. The second one binds to the negatively charged acetylheparin sulfate on the virus’s surface to prevent viral docking. Notably, no vaccines or antiviral treatments have been identified to combat whitefly fever Naples virus (SFNV) infection. However, Chianese et al. (2023) recently found that bee venom peptides can bind to acetylheparin sulfate and inhibit the SFNV virus from interacting with the cell surface, thereby inhibiting effective infection by the virus. The third type involves inhibiting viral gene expression or blocking viral enzymes or host factors involved in replication and transcription. For example, Narula et al. (2023) investigated the inhibitory effect of Tachyplesin (Tpl) on the Hepatitis B Virus (HBV). HBeAg is an early serum marker of HBV infection. Tachyplesin was found to significantly inhibit the expression of HBV proteins (HBsAg and HBeAg). In addition, Tpl inhibits HBV replication and transcription.

## 6 Main applications of AMPs

AMPs are also widely used due to their rich and outstanding biological activities. It has been applied in different fields, such as agriculture, food, cosmetics, and medicine. AMPs are currently mainly studied and applied in the medical field, and the main route of administration is local administration. Local administration not only improves the local effective concentration, but also avoids unpredictable systemic toxicity. For example, baclofenac was approved for marketing in 1948, but was later withdrawn because it was found to cause nephrotoxicity when administered orally. Topical administration was eventually adopted for wound and skin infection treatment.

### 6.1 Clinical applications

AMPs did not enter clinical trials in the decades when they were first discovered. It was not until the seriousness of bacterial resistance was found and research on AMPs was increased that AMPs gradually entered the pharmaceutical market. However, the development of clinical applications of AMPs has been slow, with most remaining in the basic research stage. AMPs entering clinical trials in 2020–2024 are shown in Table 8. The table shows that only a few drugs are being explored in clinical trials, of which only Biokiller is administered orally. As of 2024, only eight AMPs have been approved by the FDA for clinical use, namely, mycopeptides, polymyxins B and E, vancomycin, tyrothricin, short mycopeptides D and S, Rezafungin, Rezzayo, and daptomycin (Han et al., 2021; Behera et al., 2024). It is worth noting that AMPs approved for marketing tend to have a cyclic structure and are positively charged. However, daptomycin has a charge, and mycopeptides have a net charge of zero. AMPs are still primarily used as antimicrobial agents in the clinic, either as stand-alone antimicrobials or combined with conventional antibiotics for synergistic effects. Most AMPs currently in clinical studies are of animal origin, such as human, bovine, and porcine (Wang et al., 2023b).

**Table 8:** AMPs entering clinical trials in 2020–2024.

Name	Type of disease	Trial Phase	Route of administration	Clinical trials
ALRN-6924	Solid tumors, lymphomas, brain tumors	Phase I	Intravenous injection	NCT03654716
PL-18	Vaginal mycosis, bacterial vaginosis	Phase I	Vaginal administration	NCT05340790
PL-5	Diabetic foot ulcer	Phase II	Topical spray	NCT06189638
Biokiller Oral Antibacterial Gel	Periodontitis	Phase IV	Oral	NCT05530252
LEAP-2	Obesity	Phase I	Intravenous injection	NCT04621409
PLG0206	Joint infection	Phase I	Local irrigation	NCT05137314
Brilacidin	New crown pneumonia	Phase II	Intravenous injection	NCT04784897
LL-37	Melanoma	Phase II	Tumor injection	NCT02225366

PL-5 Spray is the first topical AMP spray for treating wound infections. Phase II clinical trials have shown that 2‰ PL-5 is safe and effective in the treatment of skin wound infections and has a low potential to induce antibiotic resistance (Wei et al., 2023). PLG0206 is an arginine-rich synthetic peptide that is effective against *Pseudomonas aeruginosa*. In 2022, PLG0206 enters a Phase I clinical trial to treat *Pseudomonas aeruginosa* infections in prosthetic joints (Elmassry et al., 2023). Some AMPs have also improved efficacy when administered orally during the clinical trial phase. For example, Zhao et al. (2023) administered LL-37 orally to 129 Covid-19 patients infected with the Omicron BA.5.1.3. a variant of COVID-19 to halt the progression of the virus. The results showed that LL-37 exhibited a favorable safety profile as an oral anti-COVID-19 drug and also significantly shortened the time to negative conversion of Omicron BA.5.1.3 and accelerated viral clearance.

The translation of AMPs from laboratory to clinical applications still faces many challenges. Among them, the main challenges are the high cost of AMPs development, poor stability, low bioavailability, cytotoxicity, and lack of data to evaluate the pharmacokinetics of AMPs. Currently, AMPs undergo extensive *in vitro* pharmacological experiments in the laboratory and often have favorable pharmacological effects. However, in clinical use, some AMPs cannot maintain effective therapeutic concentrations in the body, so the therapeutic effect is not ideal (Dijksteel et al., 2021). For example, Neuprex, iseganan, MSI -78, XOMA-629, Pexiganan and XMP-629, etc. have entered clinical trials. However, they failed to make further progress due to lower efficacy than controls (Zheng et al., 2025). More attention needs to be paid to the *in vivo* studies of AMPs in the future. AMPs are complicated and costly to prepare, making it difficult to achieve large-scale industrial production and thus to conduct clinical trials. Compared with antibiotics, antibiotics with equivalent efficacy are often inexpensive, which makes AMPs lack of competitiveness in the clinic. Some AMPs are toxic and hemolytic, with a narrow window between effective and toxic doses, which may pose a challenge for clinical trial design. In addition, developing additional delivery systems for AMPs and improving drug delivery methods to effectively deliver AMPs to target sites in the body are also major challenges. In the process of developing new AMPs for clinical application, we can focus on the study of AMPs with cyclic structure, amphiphilic structure and more positive charge to find the target AMPs faster. With the in-depth study of the conformational relationship of AMPs and their optimization, we believe that more AMPs will be able to be tested in the clinic and become drugs.

### 6.2 Food preservation

Foods are susceptible to bacterial contamination and spoilage during storage, processing, packaging, and transportation, so food often requires the addition of preservatives to stop the growth of microorganisms. Compared with chemically synthesized preservatives, natural preservatives are greener and safer and have attracted much attention. Some AMPs have extensive and strong killing effects on bacteria in food, and they are safe, easy to digest and absorbed by the human body. They have also become a hot spot of food additive research in recent years. Currently, the more widely studied and applied AMPs preservatives are *streptococcus lactis* and *bacillus* peptide. *Lactobacillus* peptide is a well-known bacteriocin produced by the fermentation of *Lactobacillus* and also approved by the FDA for use as a food preservative along with *ε*-Polylysine (Omer, 2024; Ruiz-Ramírez et al., 2024). *Lactobacillus* peptide has a broader range of bacteriostatic activity, is safe to consume, and is considered a natural additive. Pediocin PA-1 is also a broad-spectrum bacteriocin produced by *Lactobacillus* and has been used extensively as a food biopreservative (Barbosa et al., 2017).

In addition to adding food preservatives, we can also use antimicrobial food packaging to ensure food safety. Fortunately, AMPs can also be used in the food packaging industry through various packaging technologies, such as incorporation into active packaging systems for preserved foods. Contact with proteins or fats in the food affects its stability and antimicrobial activity, so many researchers have encapsulated it in various nanocarriers. Many studies have encapsulated AMPs in liposomes to increase their stability. For example, Cai et al. (2024) used chitosan to encapsulate ε-Polylysine into nanoliposomes (CS-EPLLP) and applied it to apple juice preservation. The results showed that CS-EPLLP enhanced the anti-*Alicyclobacillus* spp. effect of ε-Polylysine in apple juice and had no impact on the quality of apple juice. Similarly, Lai et al. (2022) synthesized an AMP (HX-12C) and a CS-HX-12C film with chitosan (CS). The results showed that HX-12C exhibited strong film-forming ability, and the formed CS-HX-12C film had good antimicrobial activity, which could be used as a good packaging material to extend the shelf life of pork.

Pathogenic bacteria, such as *Listeria monocytogenes*, can form biofilms on food processing equipment or food surfaces and can survive even in chilled environments, posing a serious threat to human health. AMPs inhibit the formation of biofilms of food pathogens and preserve a high level of stability in food preservation environments. For example, Palmieri et al. (2018) developed a novel AMP (1018-K6). 1,018-K6 was found to possess bactericidal and anti-biofilm activity against *Listeria monocytogenes* and to exhibit high structural stability over a wide pH and temperature range. Some of the AMPs may have toxic effects on host cells, and genetic modification or protein engineering could be used in the future to produce high-quality AMPs for use in the food industry. Proteases and peptidases in food can react or interact with AMPs, thus affecting the efficacy of AMPs and the quality of food. Therefore, it is necessary to select highly stable AMPs for food preservation in the future.

### 6.3 Promoting livestock growth

Adding antibiotics to feed can prevent animal diseases, promote growth and improve feed utilization efficiency. However, misuse of antibiotics in feed can lead to resistance in pathogenic microorganisms, secondary infections, and compromised safety of livestock and poultry. Therefore, antibiotics as growth promoters were banned in the European Union in 2006 (Liang et al., 2024). AMPs, as a suitable candidate for antibiotic replacement, have also gained favor in livestock and poultry farming. In particular, their application in pig and poultry farming is actively researched.

Gut health has been recognized as a significant factor affecting growth, immunity, and other production parameters in poultry (Nazeer et al., 2021). Studies have shown that AMPs improve nutrient digestibility, intestinal microbiota, intestinal morphology, and immune function activity, promoting growth in livestock and poultry (Rodrigues et al., 2021). For example, Zhang et al. (2021b) studied the effects of plectasin on yellow-feathered chickens’ growth performance, intestinal health, and immunity. The results showed that plectasin could inhibit the proliferation of harmful bacteria in the chicken intestinal tract and promote the propagation of beneficial bacteria *Lactobacillus* in the ileum, thus improving the structure of intestinal flora. In addition, plectasin could inhibit the production of pro-inflammatory cytokines in the intestine, reduce intestinal inflammation and improve feed conversion rate. Broiler *β*-defensin 13 (Gal-13) is a recently characterized defensin. Wang et al. (2023d) added Gal-13 to chicken feed, and the results showed that Gal-13 can improve intestinal digestion, antioxidant capacity and immune function, and ultimately promote the growth of broilers. AMPs can also be used against avian influenza in livestock and poultry. For example, in a recent study, Zhang et al. (2024a) found that cecropin AD was effective against H9N2 avian influenza virus (AIV) in chickens. Cecropin AD effectively inhibited the replication of H9N2 AIV in the lungs, and significantly reduced inflammatory responses and lung damage.

Although AMPs show great potential in livestock and poultry farming, they also face great challenges in application, such as high cost, low stability, low yield, and possible cytotoxicity. Most AMPs used in feed are still in the research stage and currently cannot meet the requirements of industrial applications. Only a few AMPs have been put on the market, such as Cecropin (Thacker, 2013), which is sold in China for pig diets. In the future, we can focus on the fermentation, molecular design, and optimization strategies of AMPs to enhance the biological properties and production potential of AMPs to achieve large-scale applications. It is worth noting that livestock and poultry often take feed additives AMPs orally, which requires AMPs to have good safety and stability. However, most AMPs are toxic, hemolytic, and unstable to gastric acid and digestive enzymes in the gastrointestinal tract. In addition, some AMPs are easily inactivated during high-temperature pelleting of feed (Liang et al., 2024). Nanomaterial delivery systems can effectively improve these shortcomings. Therefore, in the agricultural field, the focus should also be on improving the dosage form of AMPs to help develop more AMPs into feed additives.

### 6.4 Cosmetic application

AMPs have also been gradually applied to cosmetics because of their excellent antimicrobial activity and their lack of toxic side effects on the human body when applied externally. AMPs inhibit the growth of microorganisms in cosmetics and treat wounds and improve tissue regeneration without scarring (Alencar-Silva et al., 2018). Some AMPs researched more in cosmetics include LL-37, defensin, and others. It is well known that excessive collagen accumulation can lead to diseases such as scleroderma, scars and tumor growth. Several studies have demonstrated the ability of LL-37 to inhibit accelerated wound healing and collagen over-synthesis, thereby inhibiting the formation of scars (Park et al., 2009; Petkovic et al., 2021). Acne is a prevalent adolescent skin condition that affects one’s appearance to a greater extent, and it has attracted a great deal of attention from the cosmetic community. *Propionibacterium acnes*, a key player in the acne-producing process, resides in the hair follicles and sebaceous glands of the skin and causes various inflammatory conditions. Many studies have also shown that AMPs can inhibit the growth and inflammation of *Propionibacterium acnes* (Wu et al., 2020). For example, Theansungnoen et al. (2022) synthesized two novel AMPs (WSKK11 and WSRR11) using the methodology of the SPPS. It was found that WSKK11 and WSRR11 could effectively kill *Propionibacterium acnes* with MIC of 128 and 64 mg/L, respectively.

Currently, the application of AMPs in cosmetics is relatively small. Few studies have reported that AMPs have skin whitening, prevention or reduction of fine lines and wrinkles, and anti-aging effects, which are often points of consumer demand for cosmetics. The production of reactive oxygen species may induce skin aging. Some of the AMPs have antioxidant properties that protect against free radical damage to the skin and may be able to play an important role in anti-aging products as well. In the future, we can expand the research on AMPs in anti-aging and promote the innovation and development of AMPs in the cosmetics industry.

## 7 Conclusion and prospects

In conclusion, AMPs sources are very broad, diverse, active and widely used. Many novel AMPs have also been discovered in recent years. However, many organisms, such as marine organisms, tropical plants, and others, still need to be tapped, which are difficult to obtain. Biological materials can be obtained through diving collection, artificial cultivation, and other methods. The diverse structures of AMPs result in diverse activities and mechanisms of action. Their mechanisms of action may be unmatched by existing antimicrobial drugs. The activity of AMPs is closely related to their amino acid sequence, charge, secondary structure, hydrophobicity and amphipathicity. Pharmacophoric groups can be introduced in a targeted manner through chemical modification, and these parameters can be changed to synthesize target AMPs. AMPs are excellent candidates for therapeutic drugs because of their broad-spectrum activity, high selectivity, low toxicity and low induced resistance. Currently, they are widely used in clinical, food preservation, cosmetic and other fields, mainly for their antimicrobial activities. Especially in the clinic, it has a great potential for anti-infection, anti-tumor, promoting wound healing, becoming a vaccine adjuvant, enhancing immunity, and other fields. The mechanism of action of AMPs in exerting antimicrobial, anti-tumor, and antiviral activities is often related to the disruption of cell membranes.

As the demand for AMPs grows, technology is urgently needed to produce AMPs on a large scale. However, AMPs production efficiency is low, yield is low, cost is high, and raw materials are limited. Therefore, chemical synthesis and genetic engineering have become the main means of obtaining AMPs in large quantities. Synthetic peptide preparation is equally expensive, and the development cost is 100 ~ 500 times more costly than traditional small molecule antibiotics. Therefore, there is a need to optimize the solid phase synthesis method and automate the synthesis. It is also possible to optimize the structure of AMPs and improve their activity, stability, and selectivity through computer-aided design, molecular simulation, and other techniques. With the development of artificial intelligence technology, research on combining cell-free biosynthesis with deep learning can be enhanced, which may enable rapid and inexpensive production of AMPs. Genetic engineering is also an essential strategy for increasing AMPs production. AMPs genes are introduced into microorganisms through genetic engineering techniques, and the fermentation system of microorganisms is utilized to produce AMPs in large quantities. *E. coli* and yeast are the two main systems most commonly used to produce recombinant AMPs (Wibowo and Zhao, 2019). Recombinant DNA technology provides flexibility in the genetic engineering of microorganisms such as bacteria and yeast; therefore, this method is usually more sustainable and cost-effective than chemical synthesis. However, genetic engineering also faces great challenges in the production of AMPs. For example, AMPs may exhibit toxicity to host cells by disrupting their membrane structure. Additionally, the separation and purification process of AMPs obtained through genetic expression is often cumbersome and complicated (Wei and Zhang, 2022). Moreover, recombinantly expressed AMPs may induce immunogenic responses in the human body, so the clinical transformation of AMPs obtained by genetic engineering must meet strict regulatory requirements. With the continuous development of biotechnology, it is believed that AMPs can be produced on a large scale at a lower cost for the benefit of humanity.
